# Diversity and habitat association of medium and large mammals in Gibe Sheleko National Park, Southern Ethiopia

**DOI:** 10.1002/ece3.8000

**Published:** 2021-08-04

**Authors:** Kassahun Abie, Belete Tilahun, Abel Feyisa, Tewodros Kumssa, Alemneh Amare

**Affiliations:** ^1^ Department of Wildlife and Ecotourism Management Wolkite University Wolkite Ethiopia; ^2^ Department of Biology Kotebe Metropolitan University Addis Ababa Ethiopia

**Keywords:** abundance, distribution, diversity, mammals

## Abstract

Complete documentation on the status of mammals is indispensable for appropriate conservation measures in protected areas. However, there is inadequate information on mammalian resources in the ecosystem of Gibe Sheleko National Park (GSNP). Thus, the study aimed to assess species diversity, abundance, and habitat association of medium‐ and large‐sized mammals in GSNP. We stratified the study area into five dominant habitat types, namely dense forest, wooded grassland, grassland, riverine forest, and farmland habitat types based on land cover and vegetation structures and further employed stratified random sampling technique across each habitat type. The sample transects covered 20% of the study area. Transect width ranged from 50 m to 400 m based on vegetation cover and visibility of mammals. The main data were collected via direct observation. Data were analyzed via chi‐square test and species diversity indexes. We recorded the total of 20 mammals species' those belong to 10 families of which 8 species were large‐sized and 12 species medium‐sized mammals. There were two IUCN vulnerable species, namely *Hippopotamus amphibious* and *Panthera pardus*, and two globally near‐threatened species, particularly *Litocranius walleri* and *Caracal caracal* in the study area. Dense forest held the highest species diversity of medium‐ and large‐sized mammals (*H*′ = 2.28) with the highest evenness index (*J* = 0.84). Riverine forest had the least diversity with uneven population distribution. *Papio anubis* was the most abundance species, whereas *Caracal caracal* was the least abundant in the study area. GSNP is home for threatened and spectacular mammals species'; hence, an appropriate conservation measure is mandatory to keep existing mammals species'.

## Graphical abstract

The total of 20 species of large‐ and medium‐sized mammals was recorded in Gibe Sheleko National Park including globally threatened mammal species. Hence, imperative conservation measure is mandatory for sustainable conservation. The abundance of large‐ and medium‐sized mammals had no association with season of the year and habitat types of the study area. Dense forest habitat recorded the highest species diversity of mammals (*H*′ = 2.28) with even distribution.

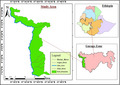

## 1 INTRODUCTION

Mammals are highly versatile group that include the fastest runners, deep divers, and most agile fliers, having colonized most of the Earth's habitats. Mammals constitute a substantial proportion of terrestrial and freshwater biodiversity (Mound & Gaston, 1993). They have a critical ecosystem functions and as a consequence must be considered in the protected area monitoring systems (Vane‐Wright, 1993; Woodroffe & Ginsberg, 1998).

Mammals are fundamental elements of most ecosystems. Large carnivores frequently shape the abundance, distribution, and behavior of prey animals (Lacher et al., 2019). Large herbivores function as ecological engineers by changing the structure and species composition of the surrounding vegetation (Owen‐Smith, 1988). Furthermore, both sets of mammals profoundly influence the environment beyond direct species interaction through cascading trophic effects (Crooks & Soulé, 1999). Large mammals perform important ecological functions and are good indicators of the habitat value because they do not typically rely on specific single habitat (Lacher et al., 2019).

Ethiopia is among the world's rich biodiversity countries with high level of endemism (Yalden & Largen, 1992). The variations in climate, topography, and vegetation have contributed to the presence of numerous endemic species. Ethiopia's high fauna diversity potential reflects the existence of many species of mammals. This in turn reflects diversity of habitats, created by the different combinations of elevation rainfall, geology, soil surface, and groundwater. For conservation of important biological resources, 27 national parks, 2 wildlife sanctuaries, 6 wildlife reserves, 25 controlled hunting areas, 5 biosphere reserves, and 8 community conservation areas have been established as refuge in different parts of Ethiopia (Tessema, 2019).

Ethiopian is endowed with 311 of mammal species belong to 14 orders of which 55 are endemic to Ethiopia. This endemism is much higher than other African countries (Lavrenchenko & Bekele, 2017). In spite of the number of mammal taxa recording for Ethiopia has been increased, still there is no complete inventory and well documentation of mammals species' in various ecosystems of Ethiopia (Tefera, 2011). However, for sufficient management and protection of protected areas, information on the status and trends of mammals is mandatory (Qufa & Bekele, 2019). The knowledge of mammals' diversity, abundance, and habitat preference is the basics for the status determination and proposing appropriate conservation measures (Gonfa et al., 2015). Moreover, determining habitat associations of mammal and environmental features important for site occupancy is quite essential to understanding the basic ecology and community organization of mammals (Stephens & Anderson, 2014). The study on species diversity and habitat association is the common tools used for ecologists and biologists in order to understand community structure and important for conservation efforts.

Gibe Sheleko National Park (GSNP) is one of the youngest protected area of Ethiopia that has been underexplored by ecologists especially on mammals species'. Hence, there is no adequate information about the mammalian resources in GSNP. To bridge these gaps, we intended to answer the following research questions: (a) what mammals species' are dwelling in GSNP, (b) how is the species diversity of medium‐ and large‐sized mammals in GSNP, and (c) is there a relationship of abundance of medium and large sized mammals with the season and habitat.

## 2 METHODS

### 2.1 Study area description

**FIGURE 1:**
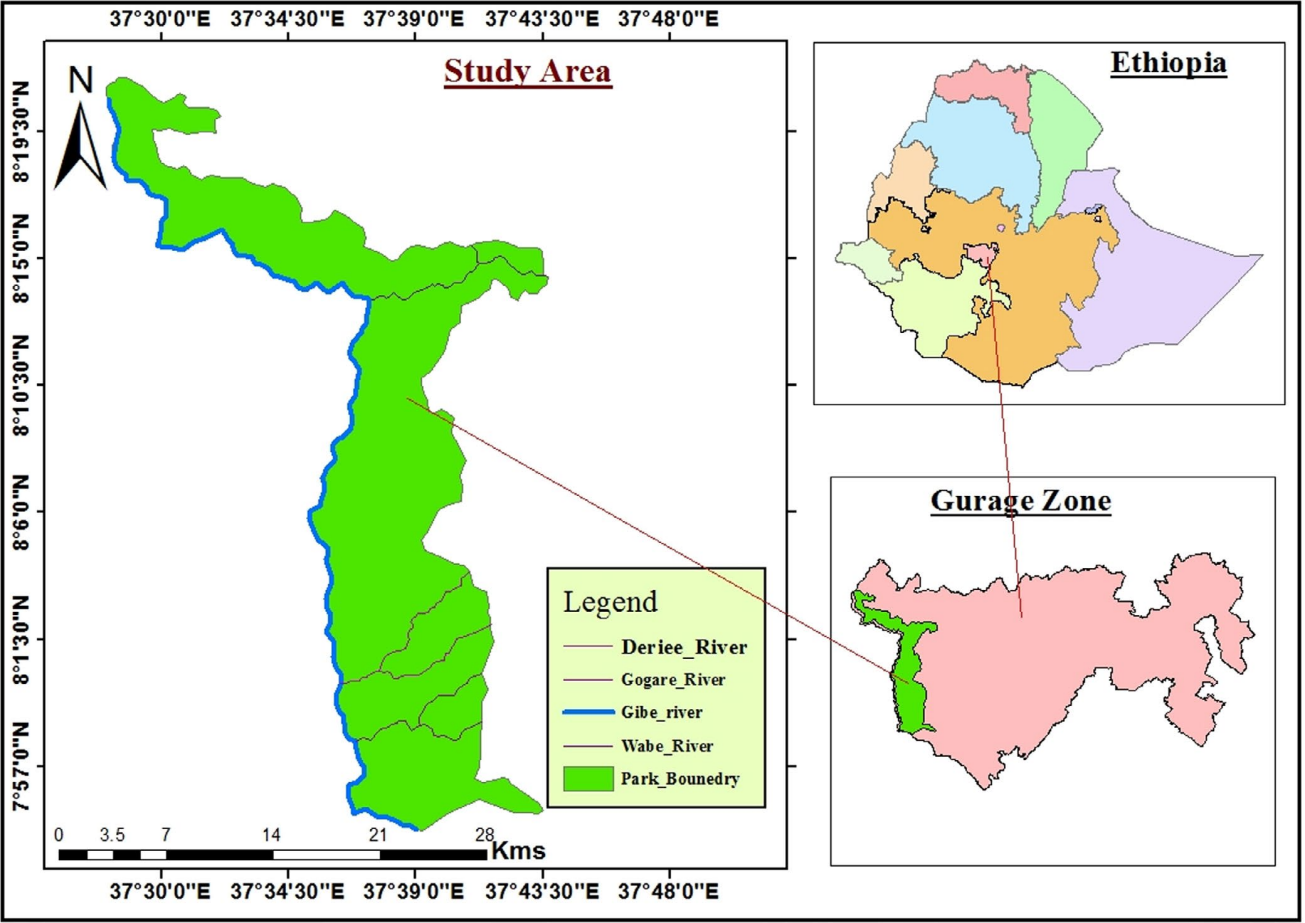
Map of the study area. Coordinate system‐Projection, Datum‐WGS 1984, UTM Zone 37N & Unit‐Meters. Source: CSA, 2007; Office of Gibe Sheleko National Park, 2009

Gibe Sheleko National Park was established as a National Park in 2009 to conserve diverse mammals and bird species. It is managed by Cultural and Tourism Bureau of the Southern Nation Nationalities Regional State. It is found 176 km far from southwest of Addis Ababa and 18 km from Wolkite town. It is geographically located between 7°54′00″ to 8°21′30″ N and 37°27′00″ to 37°45′00″E (Figure 1). It covers an area of 360 km^2^, which is characterized by heterogeneous landscape, flora, fauna, and habitat types. It is situated within three districts: Cheha, Abeshigie, and Enemurena‐Ener. Its average rainfall ranges from 960 to 1,400 mm and altitude ranges from 1,050 to 1,835 m above sea level. The temperature of the study area ranges from 10 to 28℃ (Tilahun et al., 2017). The study area is classified within the climate zone of upper kola and dissected by deep gorges of the Gibe and Wabe rivers.

#### 2.1.1 Reconnaissance survey and study period

Before the actual study commenced, a reconnaissance survey was conducted in November 2015 for 7 days to gather general information about the vegetation types, topography, and accessibility of the study area through direct field observation and interview of local people and experts of GSNP.

Data collection was undertaken from December 2015 to January 2017 during both wet and dry seasons. According to the rainfall distribution of the area, from December to April was considered as dry season and May to October was considered as wet season.

### 2.2 Study design and data collection method

The study area is characterized by heterogeneous vegetation types and topographic features. We stratified it into five dominant habitat types based on the types of vegetation structure and land cover features. These habitats include dense forest, wooded grassland, grassland, riverine forest, and farmland habitat. We classified dense forest habitat is the compacted natural forest with large canopy trees in the study area. Wooded grassland is areas covered by grasses with mix of large trees, while grassland is notoriously covered by grasses with scattered trees and twigs. Riverine forest habitat is the habitat which is covered by evergreen trees along Wabe and Gibe rivers' sides, while farmland includes human settlement and agricultural land along buffer zone of GSNP (Table 1).

**TABLE 1:** Sample area description and proportion across each habitat

Habitat types	Total area (km^2^)	Sample area (km^2^)	Habitat description
Dense forest	60	12	It is thick patches of intertwined trees which serve as agility obstacles in the forest. The habitat with thick tree and bush or closed canopy tree in GSNP. *Acacia abyssinica, Acacia nilotica, and Acacia polycantha* are the dominant tree species in the area. It encompasses from gentle slope to plain area, from 900 to 1,840 masl. Firewood and honey collection and sporadic charcoaling are among anthropogenic activities in the area
Wooded grassland	180	36	The area is covered by grass and mixture of tree species such as *Combratum cleaner, Albizzia gommifera, and Combratum molle*
Grassland	78	15.6	Grassland is the habitat that dominated by grasses. The habitat of open plains covered by grasses and sedge with very few scattered trees. Grazing and firing are commonly observing human activities in this habitat
Riverine forest	24	4.8	Riverine forest is a type of forest mostly find on the lower flood plains along the river's edge. It covers by large trees and bushes at the edge of Wabe, Gibe, Gogare, and other tributary rivers. This habitat is mostly green throughout the year including the dominant species of *Syzyguim guineense, Minusops kummel, Celtis Africana, and Ficus sycamorus*. It goes from one tip to other tip along the river's verge and from north to south and from west to east direction of the study area. Fishing and watering for livestock are often observed human interference in this habitat
Farmland	18	3.6	It is an agricultural land and settlement area along buffer zone of GSNP, mostly in Gibe‐bare kebele and Enemore site. It covers mild slop to plain area of the study area. Illegal settlement, grazing, and agricultural activities are commonly observed human pressures
Total	360	72	

The stratified random sampling technique was used via laying sample transects across the five stratified habitat types. The sample transects covered 20% (72 km^2^) of the study area. We placed transect lines proportional to the area coverage of each habitat type (Table 1) (Chanea & Yirga, 2014). The transect length was measured and located in the study area with the help of GPS (Mengesha & Bekele, 2008). We employed the total of 42 transect lines, and an average transect length ranged from 3.6 km to 8 km. Transect width ranged from 50 m to 400 m based upon vegetation cover and accessibility of the sample site (Girma et al., 2012; Girma et al., 2012). Moreover, the total number of 10, 15, 7, 5, and 5 transect lines was deployed in dense forest, wooded grassland, grassland, riverine forest, and farmland habitats, respectively. The adjacent transects were 1.5 km apart, all transect lines were roughly parallel to each other, and their ends were greater than one km far from the habitat edge to minimized edge effect (Regassa & Yirga, 2013).

We did the filed survey of medium‐ and large‐sized mammals on foot across the selected transect lines. Simultaneous transect count was deployed to avoid double counting of mammals by involving many individual observers in mammals counting the same time. We collected the data 4 times per seasons and included data collectors from wildlife expertise, ecologist, park scouts, and zoologist. All data collectors received initial training on field count, mammals' identification, and application of field materials and field guidebook (Grimmett et al., 1988; Khanum et al., 1980).

We deployed transect sample count method in the morning time from 6:30 to 10:00 a.m. and late afternoon from 5:00 to 7:00 p.m. in which most mammals were active in the study area (Brower et al., 1990; Pomeroy, 1992). The survey was carried out by careful observation with the aid of binoculars and mammals guide book particularly Kingdon (1997).

The mammals that weigh an average weight of 2–7 kg were medium and all above this were categorized as large‐sized mammals (Emmons & Feer, 1997).

Indirect detection indices such as hair samples, tracks, pellets, burrows, scratches, and other mammal's remains, for example, horn and skin, were used to know the presence and absence of nocturnal, rare, and elusive mammals species (Chanea & Yirga, 2014; Girma, Mamo, et al., 2012). We also used a key informant interview to collect general information about mammals in the area.

### 2.3 Data analysis

Data were summarized per habitat types and per season and analyzed via SPSS version 23 software. The association of mammal's abundances with the habitat types and the seasons of the year was analyzed via chi‐square test. We analyzed the relative abundance and species diversity indexes of mammals through Minitab software version 16.1.1 based on the following formulas (Table 2).

**TABLE 2:** The formulas used for data analysis

Formulas	Description
H′=‐∑pi∗lnpi	Where *H*′ is Shannon–Wiener index, *pi* is estimated as *ni*/*N*, where *ni* is the proportion of the total population of the *i*th species and *N *= ∑*ni*, this simply used proportions rather than absolute abundance values to reduce the effects of order of magnitude difference in mammals' numbers between species (Shannon & Weaver, 1949)
$J\prime =\frac{H\prime }{\ln(S)}$	Where *J*′ is Evenness index, *H*′ is Shannon–Wiener index and used the formula one, and *S* is species richness (Jarvis & Robertso, 1999; Magurran, 1988)
$\text{Relative abundance}=\frac{n}{N}*100$	Where *n* is the number of individuals of particular species recorded and *N* is the total number of individuals of the species

## 3 RESULTS

### 3.1 Species composition of medium‐ and large‐sized mammals

The total of 20 species of medium‐ and large‐sized mammals that belong to 10 families and 4 orders (Primate, Artiodactyla, Carnivora, and Rodentia) was recorded in the study area. Among the four identified orders, order Carnivora and order Artiodactyla had eight species each, and three species of order Primate and one species of order Rodentia were identified in the area (Table 3).

**TABLE 3:** Species composition, conservation status, and relative abundance of medium‐ and large‐sized mammals

Order	Family	Common name	Scientific name	CS	SM	RA (%)
Artiodactyla	Suidae	Bush pig	*Potamochoerus larvatus*	LC	Large	9.57
		Warthog	*Phacochoerus africanus*	LC	Large	9.8
	Hippopotamidae	Hippopotamus	*Hippopotamus amphibious*	VU	Large	4.86
	Bovidae	Common duiker	*Sylvicapra grimmia*	LC	Medium	1.57
		Common Bushbuck	*Tragelaphus scriptus*	LC	Large	1.36
		Kirk's dik‐dik	*Madoqua kirkii*	LC	Medium	2.56
		Waterbuck	*Kobus ellipsiprymnus*	LC	Large	3.94
		Gerenuk	*Litocranius walleri*	NT	Large	0.52
Carnivora	Felidae	Caracal	*Caracal caracal*	NT	Medium	0.31
		Leopard	*Panthera pardus*	VU	Large	0.6
		Serval cat	*Leptailurus serval*	LC	Medium	0.76
	Hyaenidae	Spotted hyena	*Crocuta crocuta*	LC	Large	1.39
	Canidae	Common jackal	*Canis aureus*	LC	Medium	1.88
		Black‐backed jackal	*Canis mesomelas*	LC	Medium	1.54
	Viverridae	African civet cat	*Civetistis civeta*	LC	Medium	1.38
	Herpestidae	White‐tailed mongoose	*Ichneumia albicauda*	LC	Medium	1.62
Primate	Cercopitheci	Olive baboon	*Papio anubis*	LC	Medium	44.32
		Vervet monkey	*Chlorocbus pygerythrus*	LC	Medium	5.2
		Colobus monkey	*Colobus gureza*	LC	Medium	4.9
Rodentia	Histeridae	Crested Porcupine	*Hystirx cristata*	LC	Medium	1.88

Abbreviations: CS, conservation status; LC, least concern; NT, near threatened; RA, relative abundance; SM, size of mammals; VU, vulnerable.

The study indicates that 8 species were large‐sized and 12 species medium‐sized mammals.

Olive baboon (*Papio anubis*) was the most abundant species (44.32%), whereas caracal (*Caracal caracal*) was the least abundant (0.31%) species in the study area (Table 3).

### 3.2 Diversity and abundance of medium‐ and large‐sized mammals

This study shows that the highest species diversity (*H*′ = 2.28) of medium‐ and large‐sized mammals was recorded in dense forest with the highest evenness index (*J* = 0.84). The least diversity index (*H*′ = 1.31) and evenness index (*J* = 0.63) were in riverine forest (Table 4).

**TABLE 4:** Species diversity and evenness index in different habitat

Habitat type	No. of species	Abundance	Diversity (*H*′)	Evenness (*J*)
Dense forest	15	844	2.28	0.84
Wooded grassland	18	1,730	1.78	0.62
Grassland	11	527	1.61	0.67
Riverine forest	9	253	1.31	0.63
Farmland	7	422	1.36	0.70

The medium‐ and large‐sized mammal's abundance was higher during wet season (121.2 ± 64.7*SE*) than dry season (67.7 ± 20.1*SE*). However, it did not show significant variation between the seasons (χ^2^ = 0, *df* = 1, *p* > .05) in the study area.

We found the highest species richness of medium‐ and large‐sized mammals in wooded grassland habitat (18 species), while farmland had the lowest species richness (7 species). The highest population proportion (45.2%) of medium‐ and large‐sized mammals found in wooded grassland habitat. The lowest proportion was (7.9%) in riverine forest during the study period. Similarly, the abundance of medium‐ and large‐sized mammals was highest in wooded grassland (89.5 ± 50.2*SE*) and the least was in riverine forest (13.3 ± 9.8*SE*) (Table 5).

**TABLE 5:** The abundance of medium‐ and large‐sized mammals across the habitat types

Species	Habitat types					
	DF	WGL	GL	RF	FL	Total
Spotted hyena (*C. crocuta)*	30	23	–	–	–	53
Bush pig (*P*. *larvatus)*	196	120	5	20	25	366
Olive baboon (*P. anubis)*	167	979	282	32	235	1,695
Vervet monkey (*C. pygerythrus)*	21	70	41	–	67	199
Warthog (*P. africanus)*	81	133	73	30	58	375
Bush Buck (*T. scriptus)*	21	19	8	4	–	52
Kirk's dik‐dik (*M. kirkii)*	58	40	–	–	–	98
Common Duiker (*S. grimmia)*	29	31	–	–	–	60
Waterbuck(*K. ellipsiprymnus)*	59	78	2	12	–	151
Common jackal (*C. aureus)*	2	30	27	–	13	72
Gerenuk (*L. walleri*)	–	15	–	5	–	20
Hippopotamus (*H. amphibious)*	–	–	–	186	–	186
Porcupine (*E. dorsatum)*	–	31	24	–	17	72
Colobus monkey (*C. gureza)*	107	70	–	10	–	187
Caracal (*C. caracal)*	12	–	–	–	–	12
Serval cat (*L. serval)*	21	8	–	–	–	29
White‐tailed mongoose (*I. albicauda)*	–	29	33	–	–	62
Civet cat (*C. civeta)*	24	20	9	–	–	53
Leopard (*P. pardus)*	16	5	–	2	–	23
Black‐backed jackal(*C. mesomelas)*	–	29	23	–	7	59
Mean ± *SE*	44.4 ± 12.9	89.5 ± 50.2	26.5 ± 14.9	13.3 ± 9.8	21.8 ± 12.7	3,824
Percentage (%)	22.07	45.2	13.8	7.9	11.03	100

Abbreviations: DF, dense forest; FL, farmland; GL, grassland; RF, riverine forest; WGL, wooded grassland.

### 3.3 Habitat association of medium‐ and large‐sized mammals

The study shows that medium‐ and large‐sized mammal's abundance had no association with all the habitats (χ^2^ = 0, *df* = 4, *p* > .05) of the study area. Nevertheless, it shows a significant difference (*p* < .05) in wooded grassland and riverine forest habitat types.

## 4 DISCUSSION

We found a total of 20 species of large‐ and medium‐sized mammals during the study period in the study area. Legese et al. (2019) reported that 12 species of medium‐ and large‐sized mammals were found in the similar ecological gradient of the study area, in Wabe forest fragment. Tilahun and Merewa (2016) reported that 19 species of mammals are recorded in Tululujia Wildlife Reserve, and Qufa and Bekele (2019) stated that 15 species of mammals are recorded in Lebu Natural Protected Forest. This dissimilarity might be due to a variation of location, area coverage, vegetation structure, composition, and the rate of anthropogenic impact (e.g., illegal settlement, agricultural expansion, and deforestation) (Qufa & Bekele, 2019).

This study reveals that two IUCN vulnerable species, hippopotamus (*Hippopotamus amphibious*) and leopard (*Panthera pardus*), and two globally near‐threatened species, particularly gerenuk (*Litocranius walleri*) and caracal (*Caracal caracal*), inhabited in the study area. The presence of rare and globally threatened species indicates that conservation importance of GSNP specifically for preservation of mammals.

Legese et al. (2019) stated that vervet monkey was the most abundant species in Wabe forest fragment. Nevertheless, olive baboon (*Papio anubis*) was the dominant species in GSNP. Likewise, Diriba et al. (2020) found that olive baboon was abundant species in Loka Abaya National Park. This is due to its flexible feeding behavior and high reproductive successes. Moreover, the species is widely distributed in variety of habitats (Johnson et al., 2012; Qufa & Bekele, 2019). Caracal (*Caracal caracal*) was the least abundant mammal species due to the habitat fragmentation, habitat loss, and degradation in the study area. Current caracal population declining in different habitats, qualifying the threshold for uplifting to near threatened status (IUCN, 2016).

According to Diriba et al. (2020) report there was a variation of species composition of mammals in different habitats. Our results also show that the highest species richness of medium‐ and large‐sized mammals recorded in wooded grassland, while the least was in farmland in the study area. High human interference (e.g., illegal settlement, considerable agricultural activities, and charcoal production) might account for this record in farmland habitat.

The highest population distribution of medium‐ and large‐sized mammals found in wooded grassland habitat and riverine forest recorded the least distribution in GSNP. The highest distribution of mammals species' in wood grassland could be due to the movement of many species from the peripheral area toward the inner in search of water, food, and cover, for example, wooded grassland is used as corridor to move to dense forest and watering point in the study area. This variation of mammals distribution also links to availability major habitat requirements (Girma, Mamo, et al., 2012; Kasso & Bekele, 2017).

The abundance of medium‐ and large‐sized mammals had no association with all habitat type of the study area, but we found a relationship of medium‐ and large‐sized mammal's abundance in two habitats in wooded grassland and riverine forest. The distribution and habitat association of mammals are often correlated mainly with the availability of water, food, and cover. Therefore, variation of vegetation structure and composition across the habitat and most mammal species use riverine area at least to get water might be contributed for this result regardless of human disturbance in the two habitats of the study area. A mammals' abundance association with their habitat is often linked to the availability of basic necessity like cover and food for animals (Chanea & Yirga, 2014; Girma, Mamo, et al., 2012; Kasso & Bekele, 2017).

Out of the five dominant habitat types, dense forest harbored the highest species diversity of medium‐ and large‐sized mammals with even distribution, whereas riverine forest had the lowest species diversity with less even distribution during the study period. However, Chanea and Yirga (2014) and Gonfa et al. (2015) reported that woodland habitat had the highest species diversity and evenness indexes. The presence of illegal agricultural encroachment and human settlement had an effect on medium‐ and large‐sized mammals obliged to stay mostly in dense forest to hide themselves. Therefore, these human interference might contribute to record the highest species diversity in dense forest habitat in the study area.

We found many skins and old and broken horns of Greater Kudu and Red Bohor from local communities during field survey. However, we could not confirm the presence of these mammals by direct and other indirect counting techniques during the study period in the area. Perhaps, they have migrated to other adjacent area or forest patch due to high illegal settlement, agricultural expansion, and deforestation in the study area.

## 5 CONCLUSION

This study reveals that GSNP is endowed with the total of 20 species of medium‐ and large‐sized mammals. Gibe Sheleko National Park harbored many IUCN Red‐listed species of medium‐ and large‐sized mammals. The occurrence of such species shows that conservation relevance of the area needs urgent conservation action in collaboration with respective stakeholders.

The dense forest supported the highest species diversity of medium‐ and large‐sized mammals with even distribution. Hence, dense forest is the most preferred habitat for most of the medium‐ and large‐sized mammals due to its better cover and foraging opportunities.

Despite many old and broken horns of Greater Kudu and Red Bohor found in the hands of local communities, no other evidence showed the presence of these mammals during the study period. This might be due to the local extinction of species from the study area or migration to other nearby forest patches of the Oromia Region. Therefore, further study shall be undertaken to confirm the presence of these species and discover other mammals in the study area.

## CONFLICT OF INTEREST

The authors declared that there is no conflict of interest.

## AUTHOR CONTRIBUTIONS

**Kassahun Abie:** Formal analysis (lead); methodology (equal); resources (equal); supervision (equal); visualization (equal); writing‐original draft (equal). **Belete Tilahun:** Data curation (lead); formal analysis (lead); investigation (lead); methodology (lead); writing‐original draft (lead). **Abel Feyisa:** Data curation (lead); investigation (equal); validation (equal); writing‐original draft (equal); writing‐review & editing (lead). **Tewodros Kumssa:** Software (equal); visualization (equal); writing‐original draft (equal); writing‐review & editing (equal). **Alemneh Amare:** Conceptualization (equal); funding acquisition (equal); investigation (equal); supervision (equal); validation (equal).

## Data Availability

All data used in the study are included in this manuscript.
